# The role of psychological flexibility in relation to health outcomes in people in remission from cancer

**DOI:** 10.1111/bjhp.12807

**Published:** 2025-06-04

**Authors:** Miznah Al‐Abbadey, Sonia Tomescu‐Stachie, Daphne Kaklamanou, Nikki Jarrett, Andrew Merwood, Ian Tyndall, Lance McCracken

**Affiliations:** ^1^ School of Psychology, Sport, and Health Sciences, Faculty of Science and Health University of Portsmouth Portsmouth UK; ^2^ Chronic Pain Service Hampshire and Isle of Wight NHS Foundation Trust Hampshire England; ^3^ School of Dental, Health and Care Professions, Faculty of Science and Health University of Portsmouth Portsmouth UK; ^4^ Macmillan Clinical Psychology Service Portsmouth Hospitals University NHS Trust Portsmouth UK; ^5^ Department of Psychology & Counselling University of Chichester Chichester UK; ^6^ Department of Psychology Uppsala University Uppsala Sweden

**Keywords:** acceptance and commitment therapy, ACT, cancer, cancer survivorship, longitudinal, psychological flexibility

## Abstract

**Objective:**

This study investigated whether psychological flexibility, the key construct in the Acceptance and Commitment Therapy (ACT) model of psychological and behavioural change, significantly predicts wellbeing and functioning in people living with and beyond cancer.

**Design:**

This was an online, prospective, longitudinal, correlational study with two time points that were approximately three months apart.

**Methods:**

All participants were required to be at least 18 years of age, be in cancer remission and resident in the United Kingdom. Recruitment for Time‐point 1 (*n* = 331) took place from May to July 2023 and Time‐point 2 (*n* = 266; 80% retention rate) took place between Sept and Nov 2023 using Prolific (an online recruitment platform). The mean age was 51.65 (SD = 13.99). The mean length of remission in months was 89.45 (SD = 80.59) and mean years since diagnosis was 8.91 (SD = 6.99). Data were analysed cross‐sectionally and longitudinally. Covariates adjusted for included age, years since diagnosis, time in remission, ethnicity, cancer type and cancer stage.

**Results:**

Cross‐sectional hierarchical regression analyses showed Time‐point 1 psychological flexibility significantly (at *p* < .001) predicted anxiety, depression, stress, fatigue interference, fear of cancer recurrence, quality of life and pain interference. Psychological flexibility at Time‐point 1 significantly predicted all psychosocial variables at Time‐point 2, while adjusting for confounding variables.

**Conclusions:**

The findings show that psychological flexibility predicts key psychosocial outcomes relevant for people in remission from cancer. This study provides evidence for the relevance of psychological processes targeted in ACT‐based interventions in the context of people living with and beyond cancer.

**Statement of Contribution**

**What Is Already Known on this Subject?**

- A growing number of cancer survivors experience significant unmet psychosocial support needs
- Evidence is emerging that supports Acceptance and Commitment Therapy (ACT) in oncology settings.
- There is uncertainty in the mechanism of change in ACT‐based interventions for cancer survivors.

**What Does This Study Add?**

- Support for psychological flexibility in predicting psychosocial outcomes in cancer survivors
- Evidence for the utility of ACT as a treatment approach for cancer survivors

## INTRODUCTION

Cancer is a leading cause of death worldwide and accounts for approximately 1 in 6 deaths globally (WHO, 2025). In the United Kingdom, one in two people are predicted to develop cancer in their lifetime, with more than half of new cases being diagnosed with breast, lung, prostate and bowel cancers (Cancer Research UK, 2024). Although the number of new cancer cases in the United Kingdom is projected to rise by 20% by 2038–2040, 10‐year survival for all cancers has doubled since 2017, with a 50% survival rate (Cancer Research UK, 2024). While it is positive that more people are surviving cancer, a growing number of cancer survivors struggle with persistent psychological and physical symptoms, including anxiety, depression, fear of cancer recurrence (FOCR), pain and fatigue (Mathew et al., 2021). Many cancer survivors experience a sense of hopelessness linked with fear of cancer recurrence, which can lead to significant mental health challenges (Andrykowski et al., 2008). Most survivors encounter some level of impact on their psychological wellbeing following a cancer diagnosis. While some cancer survivors experience growth and enhanced psychosocial adjustment, many experience a continued deterioration in emotional and physical wellbeing (Andrykowski et al., 2008). The psychological impact of cancer can extend for years after the initial diagnosis. Compared with the general population, cancer survivors report lower satisfaction with life, lower support from the community, as well as greater loneliness and lower social engagement (Proctor et al., 2023). As the number of cancer survivors, and the number of years they live, increases, the need for understanding the long‐term impact of a cancer diagnosis on mental and physical health also increases (Proctor et al., 2023).

Over 25% of cancer survivors experience a cluster of persistent physical and mental symptoms after completing treatment, including fatigue, pain, depression and sleep disturbances (Sheikh‐Wu et al., 2020; Shi et al., 2011). For example, cancer pain is complex, multifaceted, and is frequently undertreated (Russo & Sundaramurthi, 2019; Shute, 2013). At present, cancer pain is poorly understood and may be related to disease progression or the impact of medical interventions (Burton et al., 2007; Russo & Sundaramurthi, 2019). Approximately 40%–50% of cancer survivors experience pain that persists significantly beyond the completion of curative treatment (Green et al., 2011; Marshall et al., 2023). Individuals with pain report lower general health and lower physical and psychosocial functioning (Green et al., 2011). In a similar manner to pain, it is estimated that 30%–40% of cancer survivors experience cancer‐related fatigue years after treatment completion (Bower, 2014). Cancer‐related fatigue is influenced by many physiological and psychological factors (McNeely & Courneya, 2010). Psychosocial factors that have correlated with the intensity and persistence of cancer‐related fatigue include depression, anxiety, sleep disturbances and perceived social support (Brownstein et al., 2022; Kuhnt et al., 2009). In turn, cancer‐related fatigue is linked with poorer Quality of Life (QoL) and reduced psychological functioning (Bower, 2014). These findings suggest a clear need for effective, theoretically coherent and evidence‐based psychological interventions are developed to help reduce symptom burden to improve the QoL of cancer survivors.

Due to the considerable threats and direct practical impacts associated with cancer, it is understandable that the majority of cancer survivors experience high FOCR (Koch et al., 2013). While distressing, FOCR could be conceptualized as an appropriate psychological response to a life‐threatening disease such as cancer and may even function adaptively to help promote healthy adaptations (Luigjes‐Huizer et al., 2022). Despite this, FOCR has been shown to significantly contribute to both emotional distress and poorer health behaviours, including increased alcohol use and reduced physical activity (Hall et al., 2019). Managing FOCR is widely considered to be one of the most important unmet needs of people living with and beyond cancer, with over half of cancer survivors reporting at least moderate levels of FOCR (Luigjes‐Huizer et al., 2022).

A growing number of cancer survivors feel unprepared to cope with the array of physical and psychological challenges that come with survivorship, with many experiencing significant unmet psychosocial support needs (Murnaghan et al., 2024; Swash et al., 2017). Cognitive behavioural approaches appear promising for managing pain, fatigue and sleep disturbances in cancer survivors (Kwekkeboom et al., 2018; Mendoza et al., 2017; Sheikh‐Wu et al., 2020), but research is very limited. Treatments following a cognitive behavioural therapy (CBT) approach emphasize the role of cognitions in influencing maladaptive behaviours (Savard et al., 2018). For instance, this includes differentiating between ‘normal’ and ‘pathological’ expressions of FOCR (Savard et al., 2018). Although there is some support for CBT in reducing FOCR, anxiety and depression (Luigjes‐Huizer et al., 2023; Murphy et al., 2020; Park & Lim, 2022; Savard et al., 2018; van de Wal et al., 2018), evidence is rather mixed overall (van Helmondt et al., 2020). There are also limitations on the quality of evidence in terms of generalizability and variability in the implementation of cognitive behavioural interventions (Park & Lim, 2022).

Acceptance and Commitment Therapy (ACT), in contrast to CBT, rejects the view that elevated distress is necessarily abnormal or pathological (Hayes et al., 2006; Mosher et al., 2019). Instead, ACT is rooted in the philosophical stance that suffering is an inevitable part of human existence and can be better addressed by developing *psychological flexibility*, a skillset defined as ‘ability to contact the present moment more fully as a conscious human being, and to change or persist in behaviour when doing so serves valued ends’ (Hayes et al., 2006). The ACT model may, therefore, be particularly useful in the context of oncology services as patients with different diagnoses of cancer will most likely experience a significant degree of suffering that is unavoidable physically and psychologically (Fawson et al., 2023; Mosher et al., 2019, 2022; Rehfeldt & Tyndall, 2022). A number of recent systematic reviews and meta‐analyses provide emerging evidence that support ACT in improving a range of psychological factors such as anxiety, depression, psychological distress, FOCR and QoL among cancer patients or cancer survivors (Mathew et al., 2021; Sauer et al., 2024). For example, Fang et al.  (2023 found significant effects for ACT interventions on enhanced quality of life (QoL), reductions in anxiety, depression and psychological distress, and fatigue across 8 studies with a total of 488 people with advanced cancer. There were no significant effects for cancer pain or changes in psychological flexibility. Similarly, Jiang et al. (2024) conducted a meta‐analysis of 16 ACT‐based intervention randomized controlled trials that involved 711 persons with cancer and reported good effect sizes for reductions in anxiety and depression, but no significant changes for physical symptoms. In a systematic review of ACT‐based interventions in adult cancer survivors, Mathew et al. (2021) report on just 13 studies, with improvements in anxiety, depression and indicators of stress following an ACT‐based intervention. Similar to the Fang et al. ( 2023) and Jiang et al. (2024) reviews and meta‐analyses in advanced cancer patients, Mathew et al. (2021) noted the small sample sizes in the studies they reviewed and called for more larger‐scale research to determine the utility of ACT in oncology settings and whether psychological flexibility relates to better psychosocial outcomes in cancer survivors (Fawson et al., 2023). This will help provide evidence for the further development and tailoring of more effective ACT‐based interventions in the context of oncology services.

Of particular interest is to understand mechanisms of change for improvements post‐treatment in psychological wellbeing in studies that have employed ACT‐based interventions in cancer populations. For example, in their meta‐analysis, Jiang et al., 2024 calculated significant effect sizes for changes in psychological flexibility post‐ACT intervention. However, it is important to note that six of the studies that Jiang and colleagues included in their pooled effect sizes employed the Acceptance and Action Questionnaire (AAQ)‐II (i.e., Fernández‐Rodríguez et al., 2021; Ghorbani et al., 2021; Johns et al., 2020; Mosher et al., 2022; Serfaty et al., 2019; Shari et al., 2021) to measure psychological flexibility. Given the literature that suggests that the Acceptance and Action Questionnaire‐II appears to reflect psychological distress (e.g., Doorley et al., 2020; Rochefort et al., 2018; Tyndall et al., 2019; Wolgast, 2014) rather than psychological flexibility per se, there is considerable doubt that those six studies in Jiang et al., 2024 measured what they proposed and likely found instead that psychological distress was reduced by the ACT‐based intervention. This problem arises in other ACT and cancer studies in the literature that employed the AAQ‐II (e.g., Arch & Mitchell, 2016; Hulbert‐Williams & Storey, 2016; Johns et al., 2020; Kangas et al., 2015; Swash et al., 2017). Thus, to address the assessment shortcomings in the ACT and cancer literature, the present study employed measures of psychological flexibility with arguably stronger construct and discriminant validity (Ong et al., 2020), the Psy‐Flex (Gloster et al., 2021) and Comprehensive Assessment of Acceptance and Commitment Therapy Processes (CompACT; Francis et al., 2016).

### The present study

The focus of the present study is on cancer survivors. The aim of this study was to investigate the potential utility of an ACT‐based approach to support people living with and beyond cancer. To achieve this, we explored whether psychological flexibility, the key proposed mechanism of change in ACT, significantly predicts QoL, distress, FOCR, and pain and fatigue interference in people living with and beyond cancer, with a larger sample and more psychometrically sound measures of psychological flexibility than appear in the literature to date.

## MATERIALS AND METHODS

### Participants and recruitment

The inclusion criteria for the present study included participants who were over the age of 18 and have been in remission from cancer. The exclusion criteria included those not able to complete the questionnaires in English or who were not residents in the United Kingdom. We recruited participants using Prolific (Prolific, 2024), an online platform that aids in the recruitment of participants in online questionnaire‐based studies. Prolific verifies and monitors participants with sophisticated checks, without compromising data quality. Filters on Prolific were applied to target participants with cancer. Participants were paid £4.00 for each time point they completed. Participants were therefore paid a total of £8.00 for completion of the study. Payments were made automatically through Prolific. Based on a G Power calculation, we needed a minimum of 395 completed responses (this was based on a small effect size of 0.02, alpha set at 0.05 and power set at 0.80).

### Design and procedure

The study was preregistered with the Open Science Framework (registration: https://osf.io/bzdnm). The study was reviewed and approved by the University of Portsmouth (ID: SHFEC 2023–020). This was a prospective longitudinal questionnaire study with two time points that were approximately three months apart. The study questionnaires were administered online using an online survey platform (Qualtrics), which was then integrated into Prolific (Prolific, 2024).

Participants interested in taking part were required to follow a link to the study. The participants were then presented with the study information sheet, consent form, demographic questions, followed by the study questionnaires. Participants had to indicate they had read and understood the information sheet by checking the relevant boxes on the consent form before they could proceed to the study questionnaires. Participants spent on average 17 min (*SD* = 27.10) to complete Time 1 questionnaires and on average 21.40 min (*SD* = 27.10) to complete Time 2 questionnaires. Upon completion, participants were presented with a debrief sheet with links to further support should participants feel they needed this.

### Measures

#### Demographic questions

Participants reported on their age, gender, years since diagnosis, months in remission, ethnicity, cancer type, cancer stage and whether they were currently receiving treatment. Open text responses were used for reporting cancer diagnosis, date received the cancer diagnosis, length of remission, gender, age and ethnicity. Categorical response options were used for reporting stage of cancer, which ranged from staged 0 to 4 and ‘unsure’ option. Categorical options were also used to report whether participants were undergoing any current cancer treatments (‘yes’ and ‘no’ options), which was followed by the option to elaborate with open text (if they selected ‘yes’).

##### Symptom interference and intensity measures

###### **Fatigue Symptom Inventory (FSI;** Hann et al., 1998)

The FSI is a 13‐item measure that was used to measure fatigue intensity and interference. It consisted of four intensity items rated on an 11‐point scale (‘0 = not at all fatigued’, ‘10 = extreme fatigue’). It also includes a 7‐item fatigue interference subscale rated on an 11‐point scale (‘0 = no interference’, ‘10 = extreme interference’). The final two items measure fatigue duration, and the average proportion of day fatigue was present. The FSI interference subscale total score was calculated by summing the seven items within this subscale. Higher scores indicate greater fatigue interference.

###### Brief Pain Inventory (BPI; Cleeland, 1991)

The BPI is a 11‐item measure that was used to measure pain intensity and interference. It consisted of four intensity items rated on an 11‐point scale (‘0 = no pain’, ‘10 = pain as bad as you can imagine’). It also includes a 7‐item interference subscale rated on an 11‐point scale (‘0 = no interference’, ‘10 = interferes completely’). A total score for the BPI interference subscale was calculated by calculating a mean score of the seven items within this subscale. Higher scores indicate greater pain interference.

##### Wellbeing measures

###### Functional Assessment of Cancer Scale (FACT‐G) (Cella et al., 2023)

The FACT‐G is a 27‐item questionnaire that was used to measure QoL. The FACT‐G measures QoL within four dimensions including physical wellbeing, social/family wellbeing, emotional wellbeing and functional wellbeing. Items are rated on a 5‐point scale from ‘0‐Not at all’ to ‘4‐Very much’, and a total score was calculated by summing the subscales. Greater scores indicate higher QoL. The FACT‐G was selected for this study, as it has been widely used in cancer populations (Serfaty et al., 2019).

###### **Fear of Recurrence (FOR) Questionnaire** (Rogers et al., 2010)

The FOR is a 7‐item questionnaire that was used to measure fear of cancer recurrence. Items 1–6 are rated on a 1–5 scale, and item 7 is rated on a 0–10 scale. All items were summed to give a total FOR score, with higher scores indicating greater FOR.

###### **Depression Anxiety and Stress Scale (DASS‐21)** (Lovibond & Lovibond, 1995)

The DASS‐21 is a 21‐item questionnaire that was used to measure psychological distress. The DASS‐21 consists of three 7‐item subscales that measure depression, anxiety and stress. Items are rated on a four‐point scale ranging from 0 to 3. Items were summed within each subscale to calculate a total score within each domain. Higher scores indicate greater distress.

##### Psychological flexibility

Psychological flexibility was measured using two separate instruments to ensure the construct was comprehensively measured. Although the application of ACT in health settings is increasing, there is yet to be a universally agreed definition of its central construct (i.e., psychological flexibility), and previous measures have been heavily criticized due to poor reliability and discriminant validity (Cherry et al., 2021).

###### **Psy‐Flex** (Gloster et al., 2021)

Psy‐Flex is a 6‐item measure with response options that are summed and rated on a 5‐point scale ranging from 1 to 5. Higher scores on the Psy‐Flex indicate greater psychological flexibility.

###### **Comprehensive Assessment of Acceptance and Commitment Therapy Processes (CompACT;** Francis et al., 2016)

The CompACT is a 23‐item measure consisting of three subscales that measure Openness to Experience, Valued Action and Behavioural Awareness. Items within each subscale were summed to give a total score within each domain, and all items were summed to give a total overall psychological flexibility score. Higher scores on the CompACT indicate greater psychological flexibility.

### Statistical Methods

Descriptive statistics were used to report demographic information at Time‐points 1 and 2. Cronbach's alpha and McDonald's Omega were calculated to assess the reliability of each measure. There is growing criticism in the use of Cronbach's alpha as a reliability measure as its underlying assumptions are rarely met (e.g., that all items have equal variance and contribute equally to a construct; Hayes & Coutts, 2020; Malkewitz et al., 2023). McDonald's omega has been shown to be a more robust measure of reliability, particularly when there are deviations from such assumptions (Deng & Chan, 2017; Hayes & Coutts, 2020; Malkewitz et al., 2023). Therefore, both measures of reliability were used.

Hierarchical regression analyses were conducted to investigate the predictive utility of psychological flexibility scores on QoL, distress, fear of cancer recurrence, and fatigue and pain interference. Data collected at Time‐point 1 were analysed cross‐sectionally, and data collected at Time‐point 2 were merged with Time‐point 1 and analysed longitudinally. Separate hierarchical regressions were conducted including either Psy‐flex (total score) or CompACT subscale scores (Behavioural Awareness, Valued Action and Openness to Experience) using a two‐block entry method. Covariates, including age, years since diagnosis, months in remission, ethnicity, gender, cancer type, cancer stage and whether participants were receiving treatment during the study, were included in the first block. Psychological flexibility was entered in the second block to assess its added variance on the outcome variables. Hierarchical regressions were repeated to analyse the data longitudinally using a three‐block entry method. Time‐point 1 covariate data, outcome variables and psychological flexibility scores were entered in the first, second and third block, respectively. In the longitudinal analyses, outcome variables at Time‐point 2 were entered as dependent variables.

The assumptions for all hierarchical regression analyses were assessed, and in all cases, there was linearity as assessed by partial regression plots and a plot of studentized residuals against the predicted values. There was independence of residuals, as assessed by the Durbin‐Watson statistic, and all values were close to 2. There was homoscedasticity, as assessed by visual inspection of plots of studentized residuals versus unstandardized predicted values. There was no evidence of multicollinearity, as assessed by tolerance values greater than 0.1.

### Results

Participant demographic information is shown in Table 1. A total of 331 participants were recruited at Time‐point 1 with a 20% attrition at Time‐point 2. A post‐hoc power calculation was conducted using G Power, and it was revealed that the study was powered at 0.73. This calculation was based on the sample of 331, a small effect size of 0.02 and alpha set at 0.05. There were no significant differences between completers and non‐completers in gender, ethnicity, cancer diagnoses, years since diagnosis, length of remission or whether they were undergoing any cancer treatments during the study. There were also no significant differences in participant demographic between Time‐points 1 and 2. Recruited participants had a diverse range of cancer types and were at various stages. The most common cancer type was breast cancer (34.1% at Time‐point 1; 36.8% at Time‐point 2). Stages 2 and 4 were the most common cancer stages at Time‐point 1 (both 23% of participants), and Stage 2 was most common at Time‐point 2 (22.2%). The majority of participants were from a White ethnic background (93.1% Time‐point 1; 93.2% Time‐point 2). Further participant demographic information is shown in Table 1.

**TABLE 1:** Participant Demographics.

Characteristic	Time 1 (*n* = 331)	Time 2 (*n* = 266)
Age, *x̄* (SD)	51.65 (13.997)	52.78 (13.52)
Years since diagnosis, *x̄* (SD)	8.91 (6.99)	8.92 (6.54)
Months in remission, *x̄* (SD)	89.45 (80.59)	89.32 (76.25)
Gender, *n* (%)		
Female	234 (70.7)	193 (72.6)
Male	93 (28.1)	70 (26.3)
Male but assigned female at birth	1 (0.3)	0
Non‐binary	2 (0.6)	3 (1.1)
Ethnicity, *n* (%)		
White	308 (93.1)	248 (93.2)
Asian or Asian British	6 (1.8)	4 (1.5)
Black, Black British, Caribbean or African	3 (0.9)	2 (0.8)
Mixed ethnics group	8 (2.4)	5 (1.9)
Other ethnic group	5 (1.5)	5 (1.9)
Cancer type, *n* (%)		
Breast	113 (34.1)	98 (36.8)
Digestive/Gastrointestinal	23 (6.9)	17 (6.4)
Eye	1 (0.3)	0
Genitourinary	36 (10.9)	29 (10.9)
Gynaecologic	34 (10.3)	25 (9.4)
Head and neck	15 (4.5)	9 (3.4)
Blood	44 (13.3)	38 (14.3)
Musculoskeletal	8 (2.4)	6 (2.3)
Neurologic	4 (1.2)	3 (1.1)
Respiratory/Thoracic	1 (0.3)	0
Skin	37 (11.2)	27 (10.2)
Multiple cancers	14 (4.2)	13 (4.9)
Cancer stage, *n* (%)		
0	14 (4.2)	12 (4.5)
1	58 (17.5)	53 (19.9)
2	77 (23.3)	59 (22.2)
3	60 (18.1)	51 (19.2)
4	22 (6.6)	16 (6.0)
Not sure	77 (23.3)	58 (21.8)
Did not answer	23 (6.9)	17 (6.4)

Table 2 presents the descriptive statistics of all study variables at Time‐points 1 and 2. A paired‐samples *t*‐test was conducted to determine differences in study variables between time points. A statistically significant reduction in the DASS anxiety subscale was found at Time‐point 2 compared with Time‐point 1. No other statistically significant differences were found. Figures 1–13 present (see Supporting Information) spaghetti plots of individual participant scores across both time points. In contrast to the aggregate data presented in Table 2, visual inspection of the spaghetti plots suggests substantial variation in individual trajectories across the time points for each variable.

**TABLE 2:** Descriptive statistics of study variables and paired *t*‐tests of variables at T1 and T2.

Variable	Measures	Time‐point 1			Time‐point 2			Mean difference (T2‐T1)	SD	*t*	df	*P* (two‐tailed)
		T1 mean (SD)	Reliability statistics		T2 mean (SD)	Reliability statistics						
			Cronbach's Alpha	McDonald's Omega		Cronbach's Alpha	McDonald's Omega					
Psychological flexibility	CompACT ‐ OE	30.042 (10.796)	0.826	0.882	30.308 (11.074)	0.853	0.851	0.266	7.63	0.566	262	0.572
	CompACT ‐ BA	15.954 (7.262)	0.878	0.880	15.661 (7.208)	0.880	0.880	−0.293	4.72	−1.006	262	0.315
	CompACT ‐ VA	34.380 (8.148)	0.883	0.884	34.259 (8.496)	0.903	0.905	−0.122	5.14	−0.384	262	0.701
	CompACT ‐ Total	80.376 (22.203)	0.774	0.788	80.228 (22.768)	0.785	0.802	−0.148	12.65	−0.19	262	0.849
	Psy‐Flex	21.304 (4.424)	0.852	0.849	21.232 (4.679)	0.880	0.879	−0.072	3.056	−0.383	262	0.702
Pain interference	BPI ‐ interference	2.000 (2.563)	0.970	0.970	1.929 (2.570)	0.970	0.971	−0.072	1.58	−0.741	263	0.460
Fatigue interference	FSI ‐ interference	23.410 (17.201)	0.956	0.959	23.519 (17.770)	0.954	0.957	0.109	11.38	0.156	265	0.876
Distress/mental wellbeing	DASS ‐ Depression	11.318 (10.454)	0.925	0.925	11.485 (10.884)	0.939	0.940	0.167	7.098	0.382	263	0.703
	DASS ‐ Anxiety	7.727 (7.330)	0.806	0.803	7.008 (7.418)	0.822	0.822	−0.72	5.514	−2.121	263	**0.035** *
	DASS ‐ Stress	12.886 (9.466)	0.897	0.896	12.424 (9.574)	0.906	0.908	−0.462	6.626	−1.133	263	0.258
QoL	FACT‐G ‐ Emotional wellbeing	16.485 (4.447)	0.790	0.790	16.485 (4.902)	0.828	0.827	0	2.905	0	263	1.000
	FACT‐G ‐ Functional wellbeing	17.727 (6.064)	0.867	0.869	17.580 (6.034)	0.853	0.856	−0.148	3.632	−0.661	263	0.509
	FACT‐G ‐ Physical wellbeing	21.447 (5.619)	0.858	0.862	21.390 (5.638)	0.875	0.878	−0.057	2.754	−0.335	263	0.738
	FACT‐G ‐ Social wellbeing	18.159 (6.381)	0.810	0.808	18.091 (6.392)	0.816	0.812	−0.068	4.313	−0.257	263	0.797
	FACT‐G ‐ Total	73.818 (17.952)	0.805	0.814	73.546 (18.549)	0.815	0.823	−0.273	8.834	−0.502	263	0.616
Fear of recurrence	FOR	21.345 (6.643)	0.931	0.934	21.152 (7.005)	0.944	0.948	−0.193	4.018	−0.781	263	0.435

Abbreviations: BA, behavioural awareness; BPI, Brief Pain Inventory; DASS, Depression Anxiety & Stress Scale; FACT‐G, Functional Assessment of Cancer Therapy Scale; FOR, Fear of Recurrence Scale; FSI, Fatigue Symptom Inventory; OE, Openness to Experience; VA, Valued Action.

* *p* < .05.

Internal consistency for each study variable as assessed using Cronbach's Alpha and McDonald's Omega is also presented in Table 2. All reliability scores were above 0.7, indicating acceptable to excellent internal consistency across all study measures.

### Cross‐sectional analysis

Table 3 presents the correlation matrix of all primary study variables at Time‐point 1. Psychological flexibility, as measured by both Psy‐flex and CompACT, significantly correlated with all outcome variables. Data collected at Time‐point 1 were analysed cross‐sectionally, and hierarchical regression analyses were conducted to determine whether psychological flexibility significantly predicted pain and fatigue interference, distress, QoL and FOR, while controlling for participants' reported years since diagnosis, months in remission, age, ethnicity, cancer stage, cancer type or whether participants were receiving cancer treatments at the time of study. Results of the hierarchical regression analyses are presented in Table 4.

**TABLE 3:** Correlation matrix of all study variables at Time‐point 1.

	1	2	3	4	5	6	7	8	9	10	11	12	13	14	15	16
1	1															
2	.665**	1														
3	.632**	.631**	1													
4	.708**	.503**	.528**	1												
5	.796**	.882**	.835**	.792**	1											
6	.272**	.298**	.326**	.228**	.338**	1										
7	.384**	.375**	.409**	.441**	.482**	.343**	1									
8	.530**	.533**	.435**	.434**	.564**	.583**	.437**	1								
9	.520**	.461**	.457**	.550**	.580**	.636**	.519**	.616**	1							
10	.529**	.515**	.509**	.521**	.613**	.790**	.742**	.794**	.873**	1						
11	−.354**	−.452**	−.353**	−.229**	−.422**	−.286**	−.174**	−.639**	−.291**	−.409**	1					
12	−.623**	−.550**	−.583**	−.593**	−.681**	−.522**	−.442**	−.602**	−.606**	−.673**	.391**	1				
13	−.466**	−.501**	−.486**	−.317**	−.523**	−.567**	−.319**	−.584**	−.492**	−.600**	.455**	.660**	1			
14	−.568**	−.595**	−.570**	−.375**	−.618**	−.508**	−.356**	−.583**	−.474**	−.589**	.460**	.707**	.762**	1		
15	−.493**	−.457**	−.483**	−.407**	−.533**	−.730**	−.411**	−.599**	−.633**	−.734**	.347**	.659**	.637**	.662**	1	
16	−.319**	−.320**	−.311**	−.239**	−.348**	−.816**	−.359**	−.552**	−.633**	−.730**	.300**	.502**	.575**	.464**	.662**	1

*Notes*: 1‐Psy‐flex, 2‐CompACT_Openness to Experience, 3‐CompACT_Behavioural Awareness, 4‐CompACT_Valued Action, 5‐CompACT Total, 6‐Physical wellbeing, 7‐Social wellbeing, 8‐Emotional wellbeing, 9‐Functional wellbeing, 10‐Overall Quality of life, 11‐Fear of Recurrence, 12‐Depression, 13‐Anxiety, 14‐Stress, 15‐Fatigue interference, 16‐Pain interference.

** *p* < 0.01 level (two‐tailed).

**TABLE 4:** Cross‐sectional hierarchical regression analyses of psychological flexibility as a predictor of outcome variables.

Outcome variable	Psychological flexibility measure	Block	Predictor variables entered	Model				Change statistics	
				B	β	*R* ^2^	F	ΔF	Δ*R* ^2^
Pain Interference (BPI)		1	Covariates†			0.189	**2.054** **		
	CompACT	2	Openness to Experience Behavioural Awareness Valued Action	−0.028 −0.086 −0.007	−0.121 **−0.247** ** −0.022	0.292	**3.166** ***	**9.677** ***	0.103
	Psy‐Flex	2	Psychological flexibility	−0.154	−0.272	0.249	**2.798** ***	**16.337** ***	0.061
Fatigue interference (FSI)		1	Covariates†			0.207	**2.304** ***		
	CompACT	2	Openness to Experience Behavioural Awareness Valued Action	−0.319 −0.440 −0.295	**−0.207** ** **−0.191** * **−0.148** *	0.383	**4.783** ***	**19.069** ***	0.176
	Psy‐Flex	2	Psychological flexibility	−1.548	−0.415	0.349	**4.506** ***	**43.939** ***	0.142
Depression (DASS)		1	Covariates†			0.147	1.525 (ns)		
	CompACT	2	Openness to Experience Behavioural Awareness Valued Action	−0.160 −0.398 −0.450	**−0.170** * **−0.284** *** **−0.370** ***	0.551	**9.436** ***	**59.916** ***	0.404
	Psy‐Flex	2	Psychological flexibility	−1.319	−0.579	0.423	**6.174** ***	**96.607** ***	0.276
Anxiety (DASS)		1	Covariates†			0.153	**1.589** *		
	CompACT	2	Openness to Experience Behavioural Awareness Valued Action	−0.202 −0.240 −0.007	**−0.284** *** **−0.226** * −0.007	0.333	**3.835** ***	**17.998** ***	0.180
	Psy‐Flex	2	Psychological flexibility	−0.680	−0.395	0.281	**3.288** ***	**36.054** ***	0.128
Stress (DASS)		1	Covariates†			0.170	**1.812** *		
	CompACT	2	Openness to Experience Behavioural Awareness Valued Action	−0.325 −0.387 −0.015	−**0.361** *** **−0.288** *** −0.013	0.464	**6.655** ***	**36.495** ***	0.294
	Psy‐Flex	2	Psychological flexibility	−1.057	−0.485	0.363	**4.805** ***	**61.265** ***	0.193
Emotional Wellbeing (FACT‐G)		1	Covariates†			0.173	**1.848** *		
	CompACT	2	Openness to Experience Behavioural Awareness Valued Action	0.143 0.035 0.115	**0.334** *** 0.056 **0.209** **	0.393	**4.972** ***	**24.087** ***	0.219
	Psy‐Flex	2	Psychological flexibility	0.457	0.442	0.334	**4.216** ***	**48.686** ***	0.161
Functional Wellbeing (FACT‐G)		1	Covariates†			0.139	1.429 (ns)		
	CompACT	2	Openness to Experience Behavioural Awareness Valued Action	0.117 0.119 0.290	**0.203** * 0.138 **0.387** ***	0.460	**6.540** ***	**39.486** ***	0.320
	Psy‐Flex	2	Psychological flexibility	0.722	0.516	0.358	**4.690** ***	**68.730** ***	0.218
Physical Wellbeing (FACT‐G)		1	Covariates†			0.236	**2.729** ***		
	CompACT	2	Openness to Experience Behavioural Awareness Valued Action	0.04 **0.166** 0.018	0.075 **0.212** * 0.026	0.303	**3.342** ***	**6.373** ***	0.067
	Psy‐Flex	2	Psychological flexibility	0.248	0.196	0.268	**3.078** ***	**8.709** **	0.032
Social Wellbeing (FACT‐G)		1	Covariates†			0.121	1.212 (ns)		
	CompACT	2	Openness to Experience Behavioural Awareness Valued Action	0.046 **0.207** **0.216**	0.077 **0.233** ** **0.279** ***	0.333	**3.836** ***	**21.183** ***	0.212
	Psy‐Flex	2	Psychological flexibility	0.533	0.369	0.232	**2.548** ***	**29.376** ***	0.112
Overall Quality of Life (FACT‐G)		1	Covariates†			0.169	**1.801** *		
	CompACT	2	Openness to Experience Behavioural Awareness Valued Action	0.345 0.528 0.639	**0.202** * **0.208** ** **0.289** ***	0.456	**6.444** ***	**35.085** ***	0.286
	Psy‐Flex	2	Psychological flexibility	1.961	0.474	0.354	**4.622** ***	**57.885** ***	0.185
Fear of Cancer Recurrence (FOR)		1	Covariates†			0.264	**3.171** ***		
	CompACT	2	Openness to Experience Behavioural Awareness Valued Action	−0.186 −0.059 0.006	**−0.309** *** −0.065 0.008	0.367	**4.451** ***	**10.756** ***	0.102
	Psy‐Flex	2	Psychological flexibility	−0.310	−0.213	0.302	**3.633** ***	**10.754** ***	0.037

*Abbreviations*: BPI, Brief Pain Inventory; DASS, Depression Anxiety & Stress Scale; FACT‐G, Functional Assessment of Cancer Therapy Scale; FOR, Fear of Recurrence Scale; FSI, Fatigue Symptom Inventory.

^†^ Years since diagnosis, months in remission, age, ethnicity, cancer stage, cancer type, currently receiving cancer treatments.

* *p* < .05;

** *p* < .01;

*** *p* < .001.

The covariates accounted for a significant proportion of variance in pain interference, fatigue interference, stress, emotional wellbeing, physical wellbeing, overall QoL and FOCR. Psychological flexibility, as measured by both Psy‐Flex and CompACT, accounted for a significant proportion of the variance in all outcome variables (*p* < .001). Although the models were all significant overall, the beta coefficients for psychological flexibility as measured by CompACT and Psy‐Flex were not consistently statistically significant. Specifically, the Psy‐Flex was not a statistically significant predictor of any study variables on the basis of its beta coefficients. By contrast, subcomponents of psychological flexibility, as measured by CompACT, were significant predictors of a number of outcomes. Specifically, Openness to Experience was a significant predictor of fatigue interference, depression, anxiety, stress, emotional wellbeing, functional wellbeing and overall QoL. Behavioural Awareness was a significant predictor of pain interference, fatigue interference, depression, anxiety, stress, physical wellbeing, social wellbeing and overall QoL. Valued Action was a significant predictor of fatigue interference, depression, emotional wellbeing, functional wellbeing, social wellbeing and overall QoL.

### Longitudinal analysis

Table 5 presents the correlation matrix of psychological flexibility as measured at Time‐point 1 and all other study variables as measured at Time‐point 2. Time‐point 1 psychological flexibility, as measured by both Psy‐Flex and CompACT, significantly correlated with all outcome variables at Time‐point 2. Data were analysed longitudinally across Time‐points 1 and 2 using hierarchical regression analyses. This was to determine whether psychological flexibility at Time‐point 1 significantly predicted pain and fatigue interference, distress, QoL and FOR at Time‐point 2, while adjusting for covariates. Results of the longitudinal hierarchical regression analyses are presented in Table 6.

**TABLE 5:** Correlation matrix of Time‐point 1 psychological flexibility variables and all other study variables at Time‐point 2.

	1	2	3	4	5	6	7	8	9	10	11	12	13	14	15	16
1	1															
2	.665**	1														
3	.632**	.631**	1													
4	.708**	.503**	.528**	1												
5	.796**	.882**	.835**	.792**	1											
6	.276**	.292**	.301**	.249**	.332**	1										
7	.371**	.345**	.410**	.410**	.452**	.369**	1									
8	.487**	.492**	.400**	.409**	.520**	.635**	.406**	1								
9	.481**	.458**	.429**	.504**	.548**	.706**	.495**	.636**	1							
10	.497**	.487**	.478**	.489**	.572**	.829**	.725**	.804**	.879**	1						
11	−.342**	−.419**	−.309**	−.225**	−.387**	−.390**	−.170**	−.682**	−.339**	−.468**	1					
12	−.560**	−.526**	−.555**	−.551**	−.639**	−.580**	−.451**	−.661**	−.720**	−.741**	.372**	1				
13	−.440**	−.436**	−.413**	−.344**	−.473**	−.571**	−.272**	−.662**	−.564**	−.626**	.488**	.655**	1			
14	−.546**	−.549**	−.587**	−.392**	−.603**	−.545**	−.390**	−.655**	−.550**	−.652**	.494**	.701**	.740**	1		
15	−.423**	−.433**	−.422**	−.343**	−.473**	−.780**	−.389**	−.604**	−.713**	−.763**	.376**	.693**	.615**	.657**	1	
16	−.205**	−.247**	−.191**	−.207**	−.259**	−.768**	−.320**	−.517**	−.613**	−.680**	.395**	.453**	.487**	.479**	.646**	1

*Notes*: 1‐Psy‐flex, 2‐CompACT_Opennes to Experience, 3‐CompACT_Behavioural Awareness, 4‐CompACT_Valued Action, 5‐CompACT Total, 6‐Physical wellbeing, 7‐Social wellbeing, 8‐Emotional wellbeing, 9‐Functional wellbeing, 10‐Overall Quality of life, 11‐Fear of Recurrence, 12‐Depression, 13‐Anxiety, 14‐Stress, 15‐Fatigue interference, 16‐Pain interference.

** *p* < 0.01 level (2‐tailed).

**TABLE 6:** Longitudinal hierarchical regression analyses of psychological flexibility as a predictor of outcome variables.

Outcome variable	Predictor variable/psychological flexibility measure	Block	Predictor variables entered	Model				Change statistics	
				B	β	R2	F	ΔF	ΔR2
T2 Pain Interference (BPI)		1	Covariates†			0.181	**1.589** *		
	T1 CompACT	2	Openness to Experience Behavioural Awareness Valued Action	−0.036 0.026 −0.022	−0.150 0.072 −0.071	0.234	**1.906** **	**3.728** **	0.053
	T1 Psy‐Flex	2	Psychological flexibility	−0.098	−0.169	0.205	**1.758** *	**4.800** *	0.023
T2 Fatigue interference (FSI)		1	Covariates†			0.193	**1.737** *		
	T1 CompACT	2	Openness to Experience Behavioural Awareness Valued Action	−0.310 −0.500 −0.182	**−0.190** * **−0.205** * −0.088	0.337	**3.203** ***	**11.845** ***	0.144
	T1 Psy‐Flex	2	Psychological flexibility	−1.365	−0.348	0.292	**2.857** ***	**23.282** ***	0.099
T2 Depression (DASS)		1	Covariates†			0.107	0.859		
	T1 CompACT	2	Openness to Experience Behavioural Awareness Valued Action	−0.179 −0.396 −0.354	**−0.181** * **−0.266** ** **−0.279** ***	0.426	**4.618** ***	**29.969** ***	0.319
	T1 Psy‐Flex	2	Psychological flexibility	−1.290	−0.533	0.339	**3.510** ***	**57.694** ***	0.232
T2 Anxiety (DASS)		1	Covariates†			0.151	1.279		
	T1 CompACT	2	Openness to Experience Behavioural Awareness Valued Action	−0.168 −0.104 −0.110	−0.2388 −0.098 −0.122	0.281	**2.433** ***	**9.73** ***	.130
	T1 Psy‐Flex	2	Psychological flexibility	−0.634	−0.368	0.262	**2.425** ***	**24.592** ***	0.111
T2 Stress (DASS)		1	Covariates†			0.117	0.949		
	T1 CompACT	2	Openness to Experience Behavioural Awareness Valued Action	−0.281 −0.425 −0.006	**−0.316** *** **−0.318** *** −0.005	0.393	**4.037** ***	**24.584** ***	0.276
	T1 Psy‐Flex	2	Psychological flexibility	−1.021	−0.470	0.298	**2.897** ***	**42.235** ***	0.181
T2 Emotional Wellbeing (FACT‐G)		1	Covariates†			0.157	1.333		
	T1 CompACT	2	Openness to Experience Behavioural Awareness Valued Action	0.119 0.049 0.114	**0.273** ** 0.074 **0.203** *	0.343	**3.250** ***	**15.295** ***	0.186
	T1 Psy‐Flex	2	Psychological flexibility	0.474	0.444	0.318	**3.181** ***	**38.698** ***	0.161
T2 Functional Wellbeing (FACT‐G)		1	Covariates†			0.168	1.451		
	T1 CompACT	2	Openness to Experience Behavioural Awareness Valued Action	0.124 0.113 0.230	**0.221** ** 0.134 **0.320** ***	0.445	**4.995** ***	**26.921** ***	0.277
	T1 Psy‐Flex	2	Psychological flexibility	0.695	0.507	0.379	**4.166** ***	**55.566** ***	0.210
T2 Physical Wellbeing (FACT‐G)		1	Covariates†			0.266	**2.603** ***		
	T1 CompACT	2	Openness to Experience Behavioural Awareness Valued Action	0.014 0.150 0.042	0.027 **0.190** * 0.062	0.319	**2.924** ***	**4.215** **	0.053
	T1 Psy‐Flex	2	Psychological flexibility	0.234	0.182	0.293	**2.837** ***	**6.295** **	0.027
T2 Social Wellbeing (FACT‐G)		1	Covariates†			0.172	1.492		
	T1 CompACT	2	Openness to Experience Behavioural Awareness Valued Action	0.063 0.222 0.165	0.105 **0.247** ** **0.215** *	0.371	**3.670** ***	**17.029** ***	0.198
	T1 Psy‐Flex	2	Psychological flexibility	0.552	0.378	0.289	**2.774** ***	**26.879** ***	0.117
T2 Overall Quality of Life (FACT‐G)		1	Covariates†			0.197	**1.756** *		
	T1 CompACT	2	Openness to Experience Behavioural Awareness Valued Action	0.320 0.533 0.550	**0.186** * **0.206** * **0.249** **	0.444	**4.974** ***	**24.011** ***	0.247
	T1 Psy‐Flex	2	Psychological flexibility	1.954	0.465	0.373	**4.068** ***	**46.182** ***	0.177
T2 Fear of Cancer Recurrence (FOR)		1	Covariates†			0.248	**2.362** ***		
	T1 CompACT	2	Openness to Experience Behavioural Awareness Valued Action	−0.172 −0.042 0.042	**−0.283** ** −0.046 0.054	0.319	**2.914** ***	**5.621** ***	0.071
	T1 Psy‐Flex	2	Psychological flexibility	−0.278	−0.187	0.276	**2.610** ***	**6.508** **	0.029

Abbreviations: BPI, brief pain inventory; DASS, depression anxiety & stress scale; FACT‐G, functional assessment of cancer therapy Scale; FOR, fear of recurrence Scale; FSI, fatigue symptom inventory.

^†^ Years since diagnosis, months in remission, age, ethnicity, cancer stage, cancer type, currently receiving cancer treatments.

* *p* < .05;

** *p* < .01;

*** *p* < .001.

The covariates at baseline accounted for a significant proportion of variance in pain interference, fatigue interference, physical wellbeing, overall QoL and FOR at Time‐point 2. Psychological flexibility, as measured by both Psy‐Flex and CompACT at Time‐point 1, accounted for a significant proportion of the variance in all outcome variables measured at Time‐point 2.

As with the cross‐sectional analyses, each model was significant overall; however, the beta coefficients for psychological flexibility as measured by CompACT and Psy‐Flex were not consistently statistically significant. Psy‐Flex was not a statistically significant predictor of any study variables at Time‐point 2 on the basis of its beta coefficients. By contrast, subcomponents of psychological flexibility as measured by CompACT were significant predictors of a number of outcomes at Time‐point 2. Openness to Experience was a significant predictor of fatigue interference, depression, stress, emotional wellbeing, functional wellbeing, overall QoL and FOR. Behavioural Awareness was a significant predictor of fatigue interference, depression, stress, physical wellbeing, social wellbeing and overall QoL. Finally, Valued Action was a significant predictor of depression, emotional wellbeing, functional wellbeing, social wellbeing and overall QoL.

## DISCUSSION

We aimed to examine whether psychological flexibility significantly predicts QoL, distress, FOCR, and pain and fatigue interference in people living with and beyond cancer to determine the utility of an ACT‐based approach in this growing population. This was investigated both cross‐sectionally and longitudinally. Cross‐sectional data showed psychological flexibility had weak correlations with physical wellbeing and pain; weak to moderate correlations with social wellbeing, anxiety and FOCR; moderate correlations with emotional and functional wellbeing, and fatigue interference; and moderate to strong correlations with overall QoL, depression and stress. Longitudinally, baseline psychological flexibility was significantly correlated with all outcome variables measured at Time‐point 2, but the strength of relationships changed with overall QoL (moderate correlations), anxiety (moderate correlations) and fatigue (weak to moderate correlations). The results showed differential effects of psychological flexibility on the outcome variables, cross‐sectionally and longitudinally, and different processes within psychological flexibility predicted outcomes differently. This also depended on the measure used—the CompACT and Psy‐Flex performed differently in our study. Our findings suggest a level of complexity within psychological flexibility, which supports the idea that this construct is not unidimensional. Past research has also shown that different components of psychological flexibility, as measured by the CompACT, can vary in their correlations in different physical health condition samples. For instance, Proctor et al. (2023) found Openness to Experience and Valued Action were significant predictors of wellbeing and thriving in cancer survivors. By contrast, Tyndall et al. (2023) found that Openness to Experience was not a useful component of psychological flexibility within chronic illness populations.

While our findings support the notion that different components of psychological flexibility may be more relevant to different outcomes and populations, our findings also show the importance of selecting the right tool. The CompACT consistently explained a greater proportion of the variance in all models compared with Psy‐Flex. CompACT also demonstrated a more nuanced reflection of the different components of psychological flexibility, which the Psy‐Flex was not sensitive enough to test. Another key difference between the measures is that Psy‐Flex explicitly asks participants to consider a time frame when answering the items, which may indicate that it is more sensitive to contextual changes over short periods compared with the CompACT. CompACT may therefore be assessing psychological flexibility as a general response across situations rather than context‐dependent. Although Psy‐Flex was developed based on both clinical and non‐clinical populations (Gloster et al., 2021), the clinical samples were from psychiatric populations, predominantly diagnosed with major depressive disorder and anxiety disorders. It would be useful to further explore how Psy‐Flex performs in chronic illness populations, as it would be useful to have a brief measure that is context‐specific (i.e., incorporates a concrete time frame to focus responses on more recent memories) and aims to measure all six facets of psychological flexibility.

Our findings are consistent with past research which has indicated significant associations between psychological flexibility and various measures of psychological impact and functioning (Arch & Mitchell, 2016; Cherry et al., 2021). Our findings are also consistent with past research indicating that psychological flexibility is a significant predictor of depression, pain‐related and overall functioning in people living with cancer‐related pain (Duarte et al., 2023). In our study, psychological flexibility at baseline predicted pain and fatigue interference, QoL and mental wellbeing 3–4 months later. While all regression models were significant, effect sizes were particularly low for the physical wellbeing domain of QoL. Given that psychological flexibility, within ACT, is not expected to correct or fix unpleasant physical symptoms, this may not be a surprise. By contrast, effect sizes were considerably greater in other QoL domains (emotional, functional and social wellbeing) and distress outcomes (depression, anxiety and stress). This is what would be predicted with greater psychological flexibility as it enables individuals to live with unpleasant or distressing experiences, which may lead to higher QoL (Hulbert‐Williams et al., 2015).

Although we did not conduct mediation analyses, our findings align with past research that has shown a mediating role of psychological flexibility in clinical outcomes, including emotional difficulties and QoL following ACT‐based interventions in oncology settings (González‐Fernández & Fernández‐Rodríguez, 2019; Mathew et al., 2021). There are very limited studies that have explored psychological flexibility (the target construct of ACT‐based interventions) in cancer survivors using non‐experimental designs, which are essential in developing theoretical frameworks in which interventions could be based on Hulbert‐Williams and Storey (2016). Past explorative research with cancer populations have reported moderating effects of psychological flexibility between unmet psychosocial care needs and psychological distress (Swash et al., 2017) and that specific components of psychological flexibility (namely openness to experience and valued action) significantly predicted wellbeing and thriving (Proctor et al., 2023). However, such findings have been based on cross‐sectional designs and our study has considerably advanced upon prior research by exploring the predictive utility of psychological flexibility on a wide number of psychosocial outcomes longitudinally.

Despite the growing body of literature in this field, the specific, dynamic, incremental, temporal processes through which components parts of psychological flexibility influence key outcomes for individuals living with and beyond cancer remain unclear (Hulbert‐Williams & Storey, 2016). This is important as it would be useful to understand the function of psychological flexibility and the process of change following a cancer diagnosis and the psychosocial implications of this. This will help determine a clearer theoretical basis for ACT‐based interventions for cancer survivors. Findings from many previous studies are based on single‐arm studies, feasibility or preliminary study designs with small samples, and many have not assessed adherence to the treatment protocols (Arch & Mitchell, 2016; González‐Fernández & Fernández‐Rodríguez, 2019; Han et al., 2019; Mathew et al., 2021; Serfaty et al., 2019), which limit the conclusions that could be drawn from such designs. In the current study, individual and aggregate trends for each psychosocial outcome were explored, and there was substantial variation in individual trajectories across the time points for each variable. Although we did not test the ergodicity of the data, visual inspection of the trends would suggest that the average trends do not represent individual changes that well. Therefore, it may be more useful to utilize more specialized N‐1 or case‐series designs to fully appreciate the complexity of change within the cancer survivor population, as relying on averages may oversimplify trends and conclusions (Gloster et al., 2024). Such idiographic approaches will also support the advancement of individualized therapeutic approaches by exploring individual processes of change.

### Study strengths and limitations

A challenge with exploring the psychological flexibility as a mechanism relates to a lack of a universally agreed upon definition and inadequate measures for the construct (Cherry et al., 2021). The AAQ‐II (Bond et al., 2011), a widely used measure of psychological flexibility, has been strongly criticized for poor discriminant validity and may be more of a measure of distress (Tyndall et al., 2019). Many studies exploring ACT in oncology have relied on the AAQ‐II (or a version of this scale) to measure psychological flexibility (Arch & Mitchell, 2016; Hulbert‐Williams & Storey, 2016; Johns et al., 2020; Kangas et al., 2015; Swash et al., 2017). It is vital for key constructs in psychological research to be operationalized accurately with universally accepted definitions. To improve our study's internal validity, we utilized both the Psy‐flex and CompACT to measure psychological flexibility as they each measure core elements of psychological flexibility (Francis et al., 2016; Gloster et al., 2021). The CompACT consistently explained a greater proportion of the variance in all regression models. The design of our study is also a strength as we implemented a longitudinal approach to collect data. This enabled us to explore trends in our study variables over two time points, which strengthens our study findings. Moreover, we were able to recruit a diverse sample in terms of the range of cancer types and stages.

There are a number of limitations to consider in relation to our findings. Although we managed to achieve an 80% retention between Time‐points 1 and 2, we were still underpowered and a minimum of 395 completed responses was required for adequate power. In addition, the current study only included two time points, which were only approximately 3 months apart. The study population had been in cancer remission for an average of 89 months (or approximately 7 years), and so, the three‐month period may have been quite a short time period relative to the amount of time the participants had been in remission. Although time in remission had been included as a covariate in the regression analysis, findings may be different if a limit was set on the time in remission or years since initial diagnosis (Hulbert‐Williams & Storey, 2016). Primary study variables, including both outcomes and psychological flexibility, did not significantly differ on average across the two time points. The two time points in this study may not have been able to capture complex patterns of change. It would be beneficial to explore psychological flexibility and how this may change from diagnosis over a longer time span in cancer survivors and how this relates to psychosocial wellbeing, QoL and functioning. This would provide further evidence for when would be best to implement ACT‐based interventions and perhaps support targeting negative psychological symptoms at earlier stages of survivorship (Mathew et al., 2021). Finally, our sample was limited in its ethnic and gender diversity and consisted of participants predominantly from a white ethnic group and mostly female. The online nature of recruitment also meant that we were unable to verify self‐reported diagnoses, and our recruitment may have skewed towards a younger demographic. Future research could explore the role of psychological flexibility across diverse populations and target clinical populations.

## CONCLUSION

Psychological flexibility was found to significantly relate to QoL, distress, FCOR, and pain and fatigue interference. This relationship was significant cross‐sectionally and across two time points that were 3–4 months apart. The findings add to the evidence base in providing further support for psychological flexibility as a target construct in interventions, for example, ACT‐based interventions in cancer survivors. Further research is needed to understand the role of the particular component parts within psychological flexibility as a set of mechanisms (see Assaz et al., 2023) and the outcomes that are expected to improve within the cancer population.

## AUTHOR CONTRIBUTIONS

**Miznah Al‐Abbadey:** Conceptualization; formal analysis; supervision; writing – original draft; funding acquisition; writing – review and editing; project administration; methodology; data curation; resources; visualization. **Sonia Tomescu‐Stachie:** Data curation; writing – review and editing; project administration; investigation. **Daphne Kaklamanou:** Conceptualization; funding acquisition; writing – review and editing; methodology. **Nikki Jarrett:** Conceptualization; methodology; funding acquisition; writing – review and editing. **Andrew Merwood:** Conceptualization; methodology; funding acquisition. **Ian Tyndall:** Writing – review and editing; conceptualization; methodology; funding acquisition. **Lance McCracken:** Supervision; conceptualization; methodology; funding acquisition; writing – review and editing.

## Supporting information


Figures S1–S13.

## Data Availability

The data that support the findings of this study are available from the corresponding author upon reasonable request.
