# Supplementary material for: The role of psychological flexibility in relation to health outcomes in people in remission from cancer
Source: Br J Health Psychol. 2025 Jun 4;30(3):e12807. doi: 10.1111/bjhp.12807 (PMC12138322; doi:10.1111/bjhp.12807)

**Figure 1**

*Individual vs aggregated trends for Psy-Flex scores at Time-point 1 and Time-point 2*


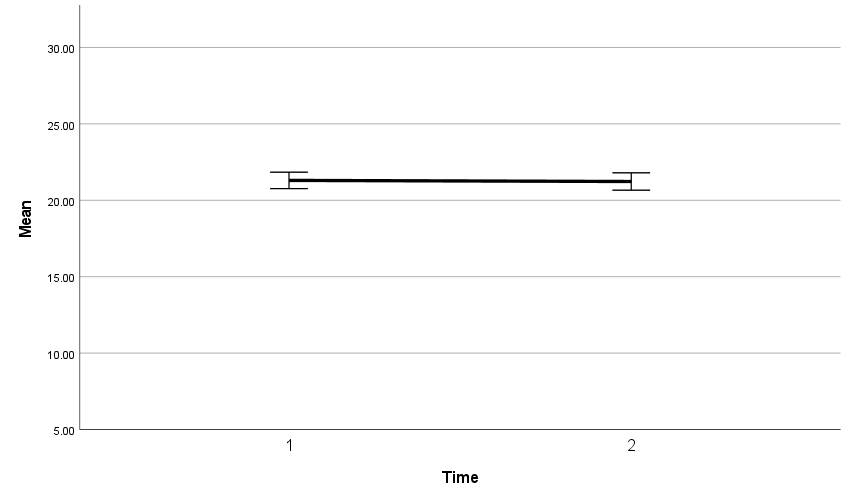


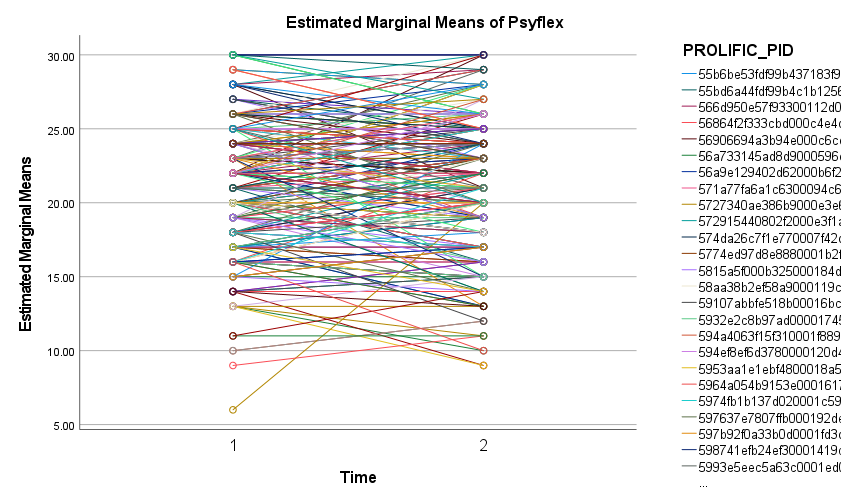


**Figure 2**

*Individual vs aggregated trends for CompACT scores at Time-point 1 and Time-point 2*


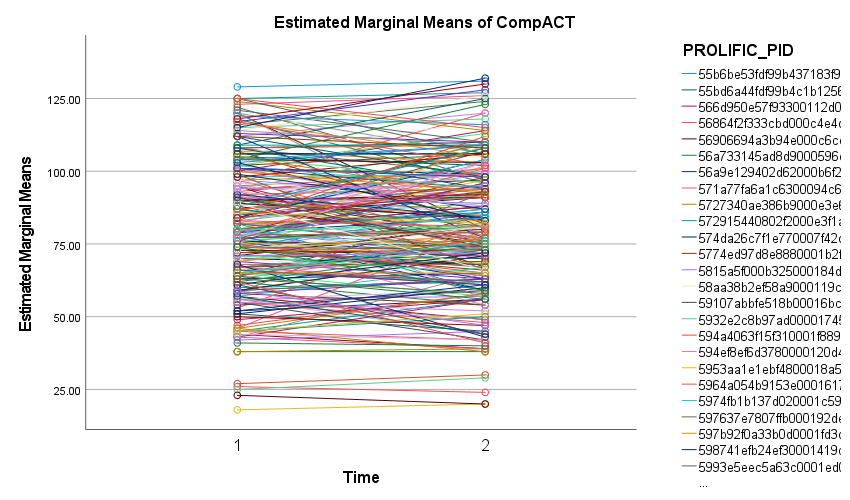

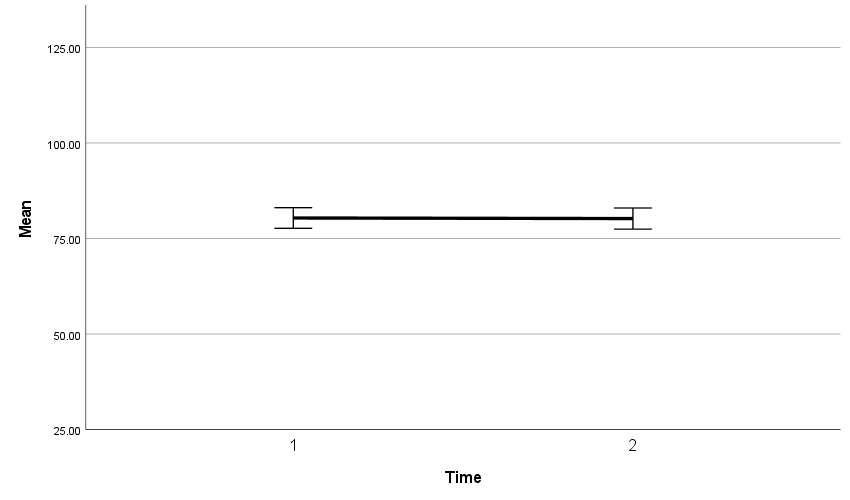


**Figure 3**

*Individual vs aggregated trends for FACT-G physical wellbeing subscale scores at Time-point 1 and Time-point 2*


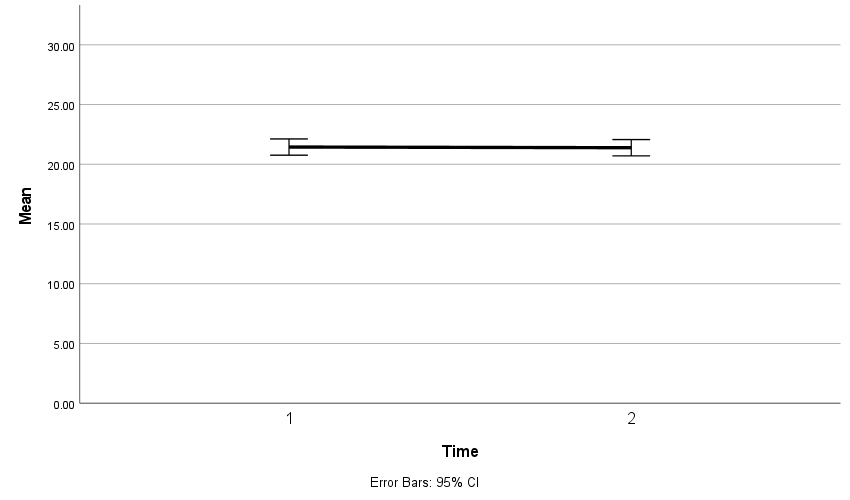

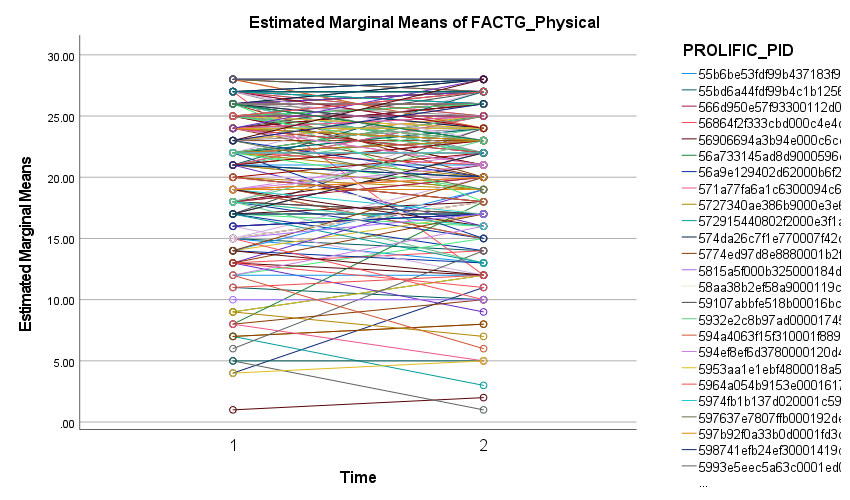


**Figure 4**

*Individual vs aggregated trends for FACT-G social wellbeing subscale scores at Time-point 1 and Time-point 2*


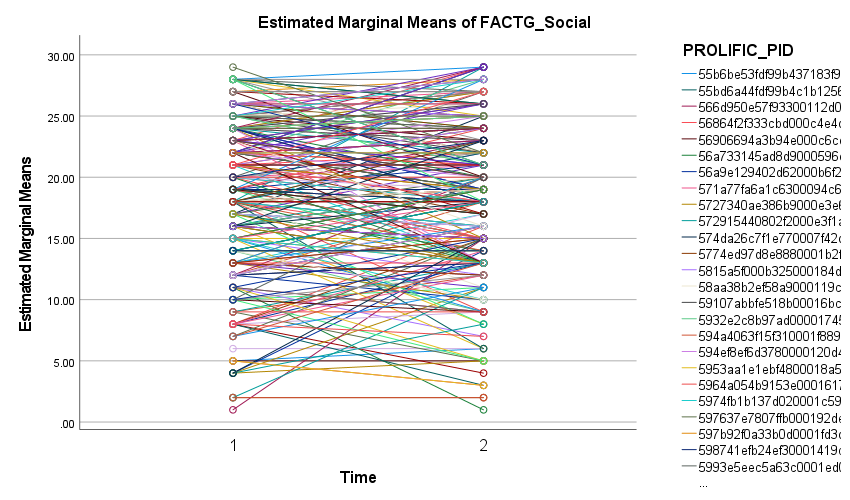

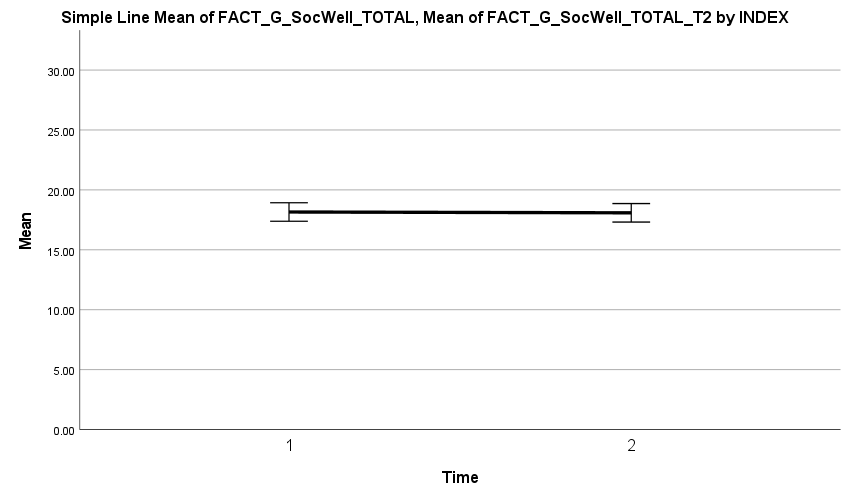


**Figure 5**

*Individual vs aggregated trends for FACT-G emotional wellbeing subscale scores at Time-point 1 and Time-point 2*


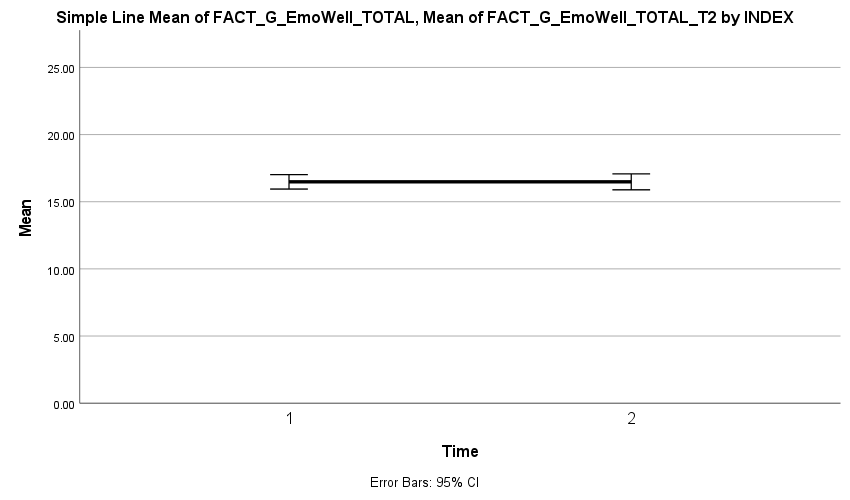

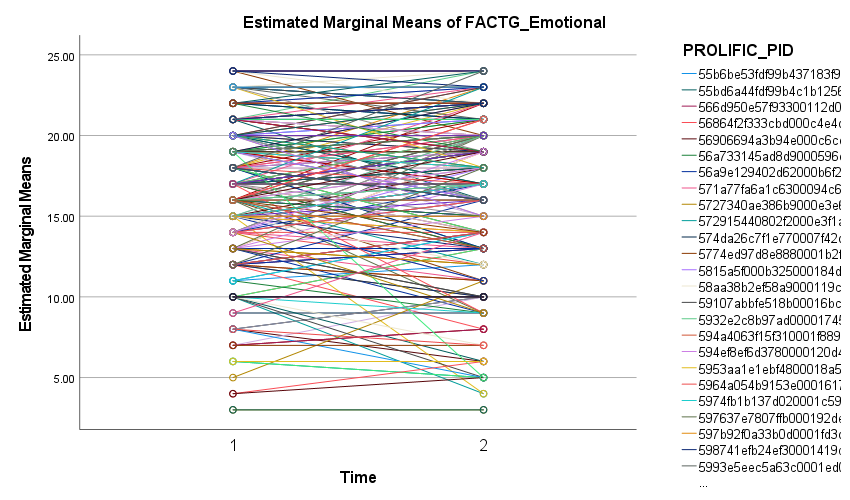


**Figure 6**

*Individual vs aggregated trends for FACT-G functional wellbeing subscale scores at Time-point 1 and Time-point 2*


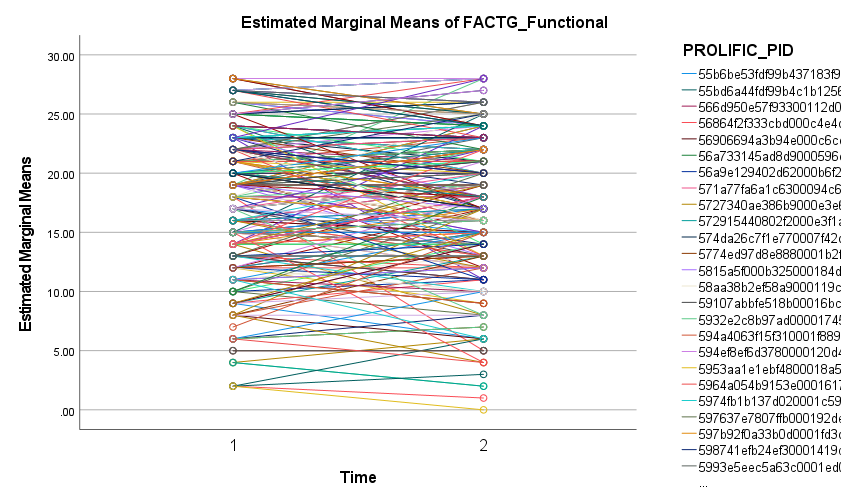

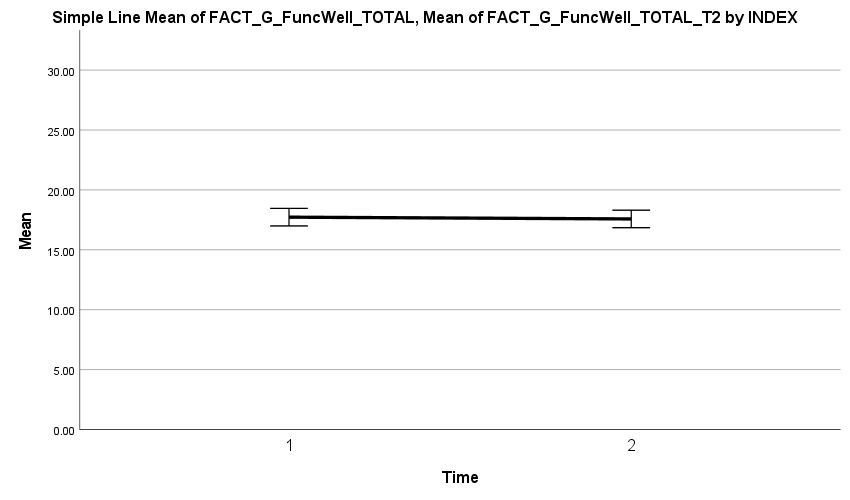


**Figure 7**

*Individual vs aggregated trends for FACT-G total scores at Time-point 1 and Time-point 2*


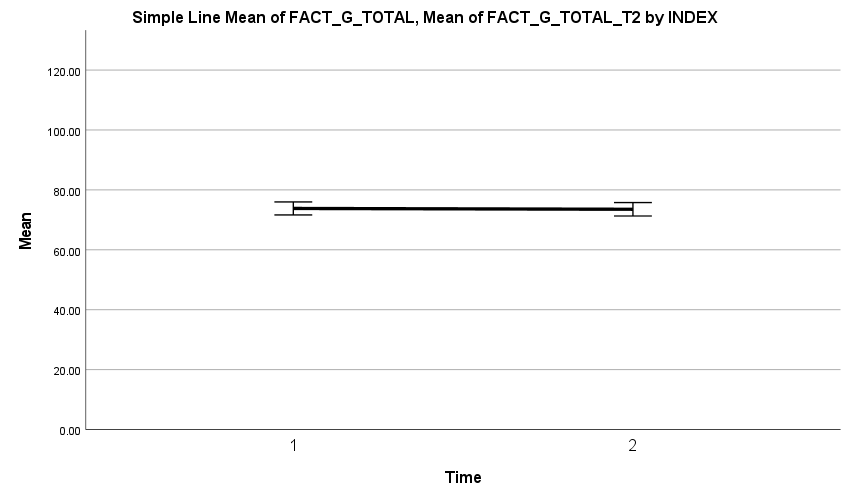


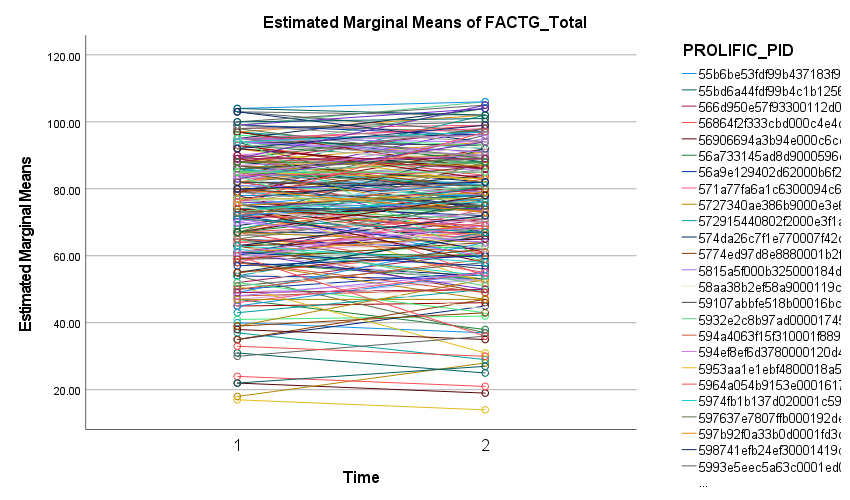


**Figure 8**

*Individual vs aggregated trends for Fear of Recurrence scores at Time-point 1 and Time-point 2*


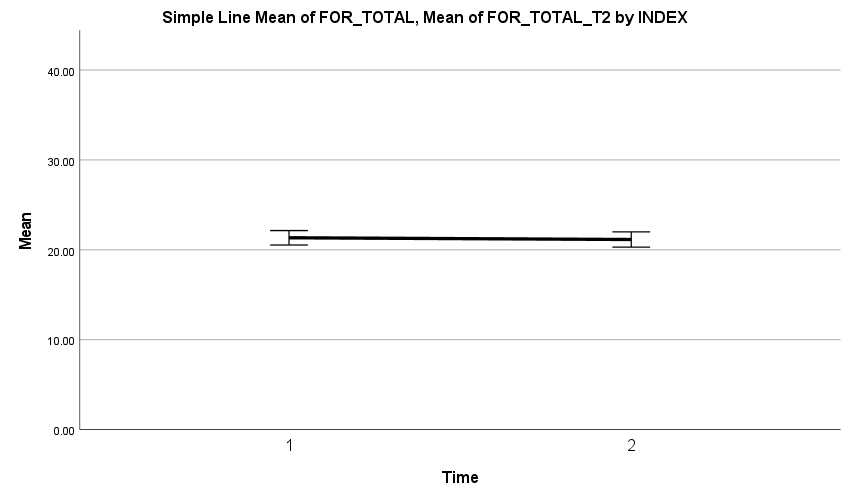


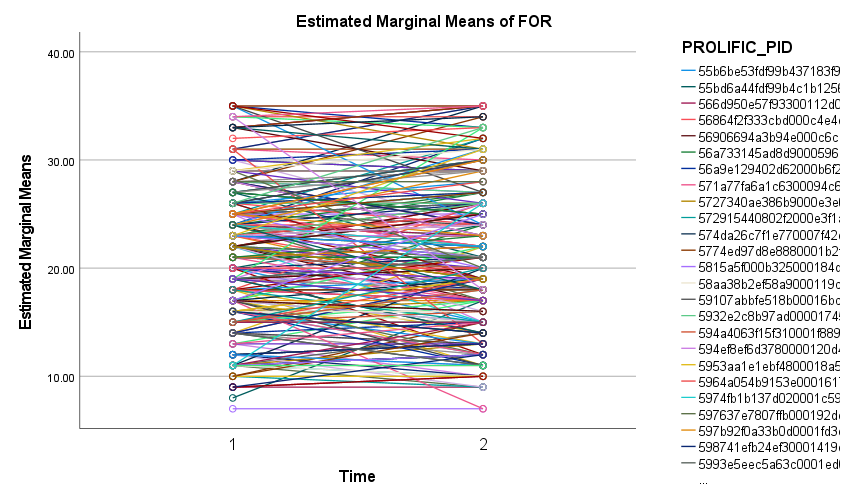


**Figure 9**

*Individual vs aggregated trends for Depression scores at Time-point 1 and Time-point 2*


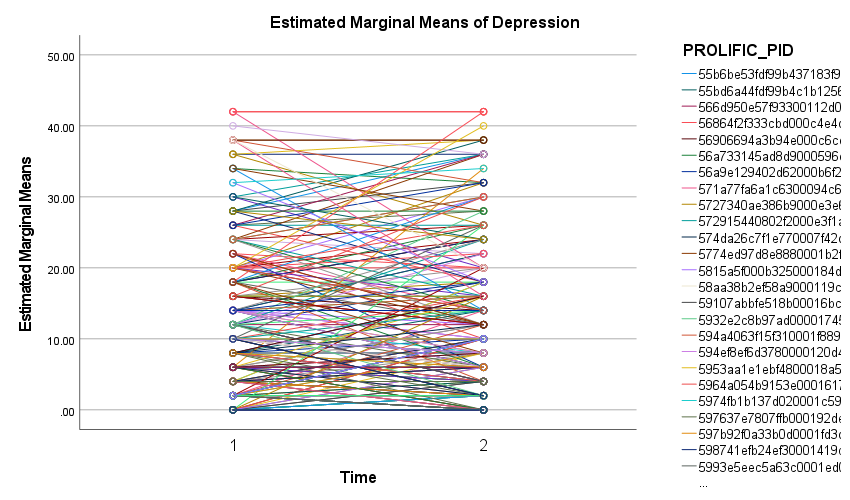

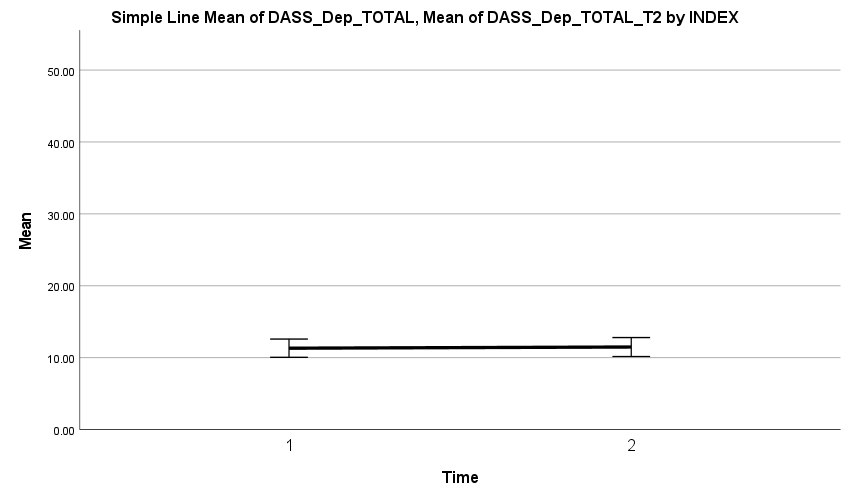


**Figure 10**

*Individual vs aggregated trends for Anxiety scores at Time-point 1 and Time-point 2*


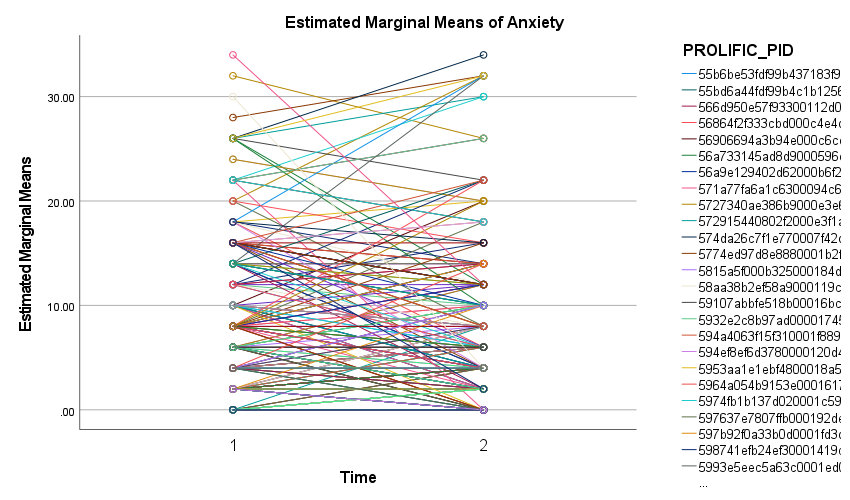


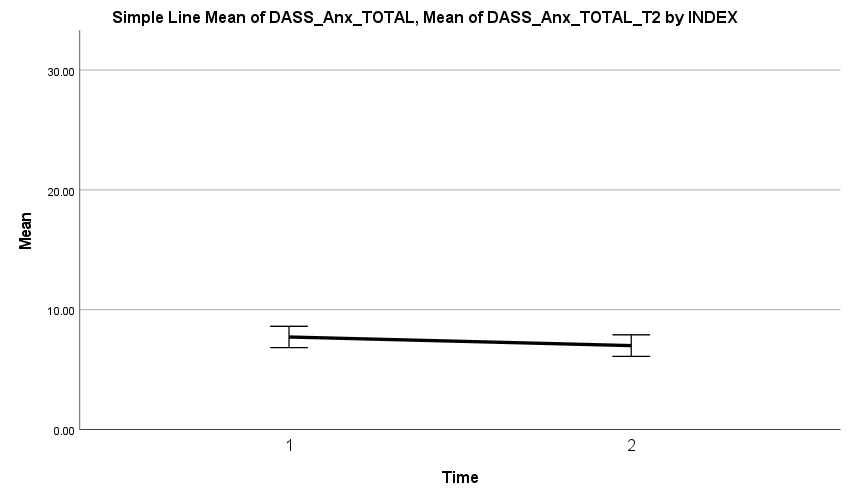


**Figure 11**

*Individual vs aggregated trends for Stress scores at Time-point 1 and Time-point 2*


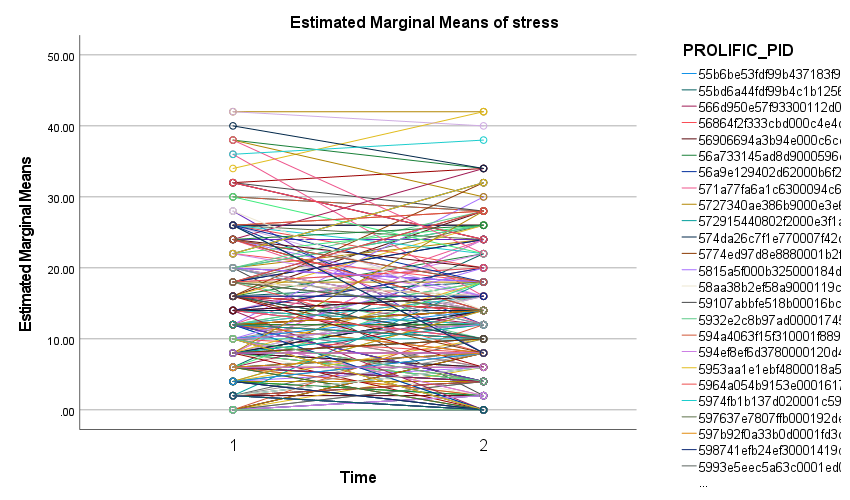

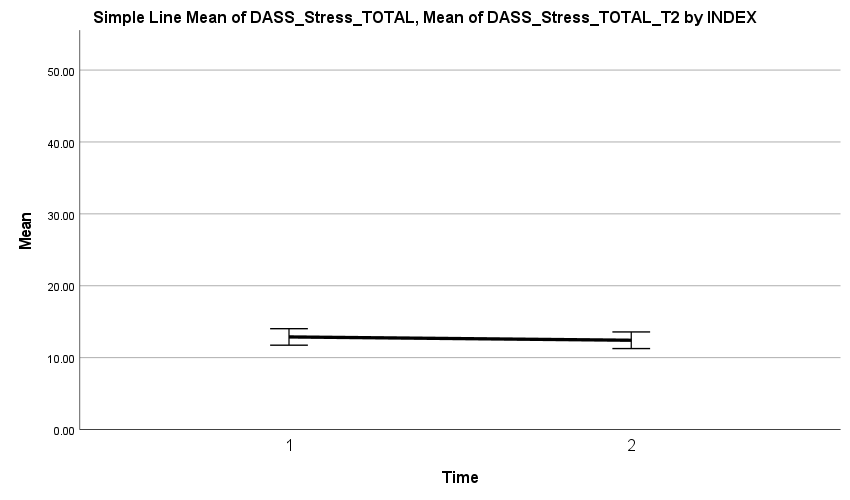


**Figure 12**

*Individual vs aggregated trends for Fatigue Interference scores at Time-point 1 and Time-point 2*


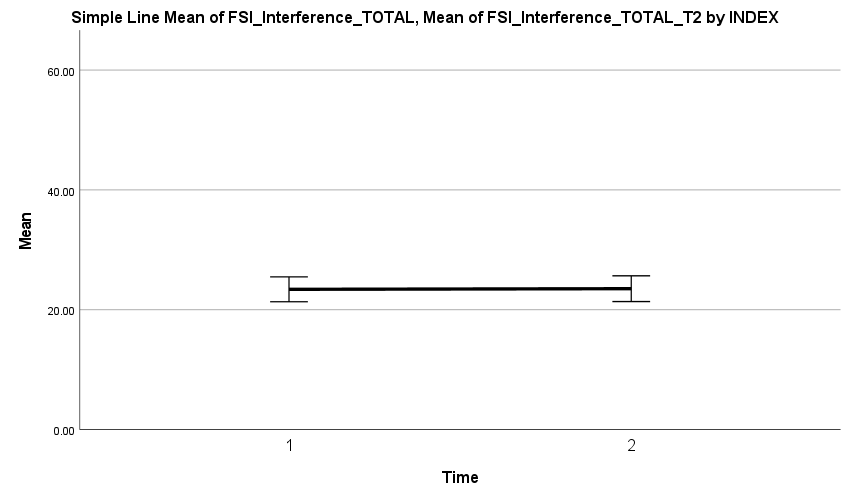

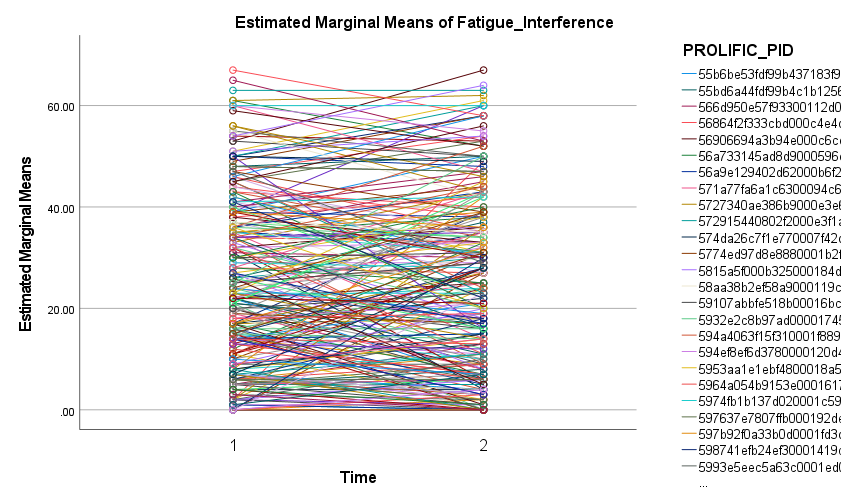


**Figure 13**

*Individual vs aggregated trends for Pain Interference scores at Time-point 1 and Time-point 2*


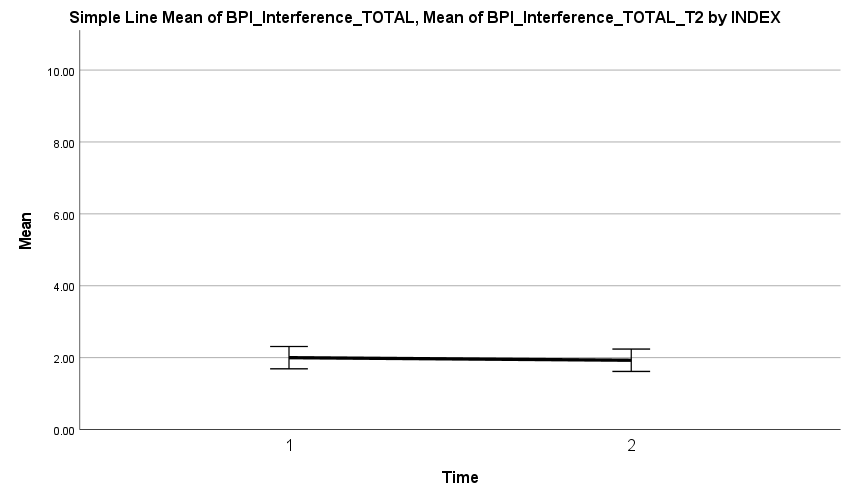


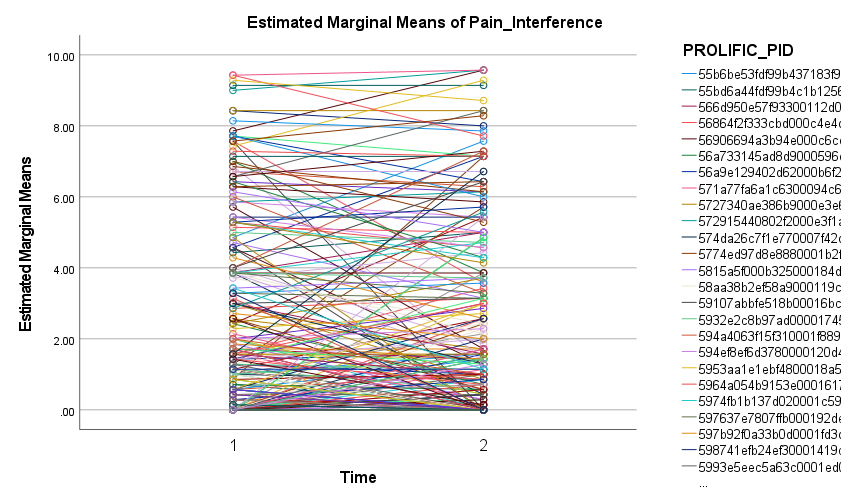

Supplement: Supplementary file 1 — Figures S1–S13. [file BJHP-30-0-s001.docx]
